# Fighting healthcare rocketing costs with value-based medicine: the case of stroke management

**DOI:** 10.1186/s12913-020-4925-0

**Published:** 2020-02-01

**Authors:** Federico Esposti, Giuseppe Banfi

**Affiliations:** 1 Università Vita-Salute San Raffaele, via Olgettina 58, Milan, Italy; 2 IRCCS Ospedale San Raffaele, via Olgettina 60, Milan, Italy; 3 IRCCS Istituto Ortopedico Galeazzi, via Galeazzi 4, Milan, Italy

**Keywords:** Value-based medicine, Cost reduction, Efficiency in healthcare, Stroke

## Abstract

Value-Based Medicine (VBM) is imposing itself as 'a new paradigm in healthcare management and medical practice.

In this perspective paper, we discuss the role of VBM in dealing with the large productivity issue of the healthcare industry and examine some of the worldwide industrial and technological trends linked with VBM introduction. To clarify the points, we discuss examples of VBM management of stroke patients.

In our conclusions, we support the idea of VBM as a strategic aid to manage rising costs in healthcare, and we explore the idea that VBM, by establishing value-generating networks among different healthcare stakeholders, can serve as the long sought-after redistributive mechanism that compensate patients for the industrial exploitation of their personal medical records.

## Background

Population ageing, rise in prevalence of chronic diseases and ever-improving medical technologies, drugs and standards are major stressors for the sustainability of healthcare systems [1]. Under such conditions, the worldwide healthcare industry is exposing its main Achille’s heel: inefficiency.

In similar-size worldwide industries, such as IT manufacturing of laptops and cell phones, innovation of technology, economies of scale and raising of operation efficiency allowed a substantial increase in performance over the last 30 years, together with a drastic reduction in prices (about 75 and 85%, respectively [2]). In the same way, in the last 20 years, the labour productivity of the healthcare workforce increased of a scant 6%, as compared to the 18% of other service industries and the 78% of manufacturing [1].

The results of such inefficiency, coupled with the aforementioned stressors, are spiralling costs and large variations in outcomes, even among hospitals of the same country [3]. At the systemic level, this situation leads to a medical inflation that in all OECD countries largely exceeds GDP growth [1, 3], together with budget reductions, declining reimbursement, resource constraints, and difficulties in delivering high standards of care based on increasingly expensive medical interventions.

The future sustainability of healthcare systems requires fast and profound improvements in efficiency and productivity of the healthcare industry. As for every other service industry, this process goes through a maturation of the relationship with the patient/customer, in which outcomes/products are defined based on the patients’ priorities. Along this line, while pursuing efficiency in healthcare has been traditionally interpreted as trivial cost cutting, more recently, the paradigm shifted toward maximization of *value* produced by medical interventions [4].

## Main text

### Value-based medicine

Centring medical practice around *value* it means defining the worthiness to pay a medical performance based on its potential to achieve a clinical, social or financial outcome [4, 5]. The idea behind this approach postulates that an optimal use of resources can be obtained by pursuing the outcomes the patient values the most, rather than focusing on cost-cutting per-se [6]. In other words, value-based medicine, VBM, is a redefinition of patient-centred targets for healthcare strategic interventions and optimization policies, reducing the risk of false economies linked with cost-reductions designed on poorly defined objectives [4].

According to our analysis, at a global level the pursuit of defining and building *value* for VBM is leading to five well-defined trends.

#### 1. Establishing policies for value-based pricing and reimbursement

The maximization of medium- and long-term outcomes is driving institutional and private payors at establishing value-based pricing and reimbursement policies. In practical terms, this means a progressive transition from procedure-payments to episode-payments, fostering the integration of nowadays-fragmented clinical pathways in integrated ones, encompassing both pre-admission and post-discharge services [7]. As an example, Germany (from 2000) and the Netherlands (from 2010) introduced such a system for various chronic diseases as diabetes and chronic obstructive pulmonary disease, which are endowed with complex and long-term post-discharge services [8]. A similar transformation is underway in drug reimbursement schemes, where an indication value-based pricing (IBP) framework is gaining popularity [9] across US and some European Countries, e.g. Italy. In this case, the reimbursement is proportional to the *value* a molecule has for each specific pathology, and it is not defined according to standard volume-price logics [9, 10].

#### 2. Defining patient-centric outcomes

The search for a proper and patient-oriented definition of *value* is leading to the creation of a number of different approaches to measure outcome. One of the most widespread and promising is the definition of Patient Reported Outcome Measures (PROMs) [5, 11] and Patient Reported Experience Measures (PREMs). These calculate health and wellbeing gains using pre- and post-intervention surveys [5], encompassing caregiver-defined (PROMs) or patient-defined (PREMs) questions/issues. In this way, patients and their informal caregivers are included in the development of the outcome review, measuring the procedure efficiency based on the patients’ priorities [12]*.*

#### 3. Data-driven operations in healthcare

The need for sophisticated information about patients’ medical history and short-, medium- and long-term clinical and social outcomes is promoting an IT revolution in hospitals. These changes aim at making all clinically relevant data manageable and available, and at allowing integration between institutional and individual data. As healthcare delivery will involve and require more and more data and services both before admission and after discharge, hospitals will need to organize an IT infrastructure able to foster the role of hospitals as hubs for preventive, primary, acute and follow-up healthcare. This revolution is forcing hospitals to invest in interoperability, standardization and connectivity [5], and it is driving investments toward the healthcare service industry for the implementation of patients’ remote monitoring, remote consultations, cloud computing and data analytics solutions [5, 13]. In its most extreme forms, the transformation of hospitals into hubs is leading to the creation of totally decentralized hospitals, i.e. fully virtual healthcare services, such as the Indian *Apollo* virtual hospitals or the *Ningbo* and the *Evergrande Group* cloud hospitals in China [13]. According to recent projections, 10% of the world’s hospitals will become, or will be in the process of becoming, “smart” by 2025 [14].

#### 4. Artificial intelligence and big data

Together with an IT revolution, the intrinsically complex nature of VBM data (which frequently are fragmented and non-structured by definition), coupled with the aforementioned effort in achieving efficiency in healthcare, is driving an unprecedented level of employment of Artificial Intelligence (AI) and Big Data Mining analysis techniques to clinical data [15–20]. AI approaches are tested and employed at all levels of the clinical pathway, before diagnosis [21], for diagnosis [22–39], for treatment and prognosis (treatment- and hospitalization [33, 40, 41], complications [27, 42–46], susceptibility to infections and relapses forecast [30, 47]) and for remote home monitoring after discharge [14, 16, 48]. At the same time, Big Data mining is increasingly employed on the already-existing clinical data mass, allowing all healthcare stakeholders to ameliorate efficiency in terms of variability reduction [49], treatment personalization [37, 50–52], identification of patterns or side effects in responses to treatments and relapses [18, 42, 53], admission and readmission rates to ERs [41, 54–56], and overall medical output at discharge [57].

#### 5. Value-based procurement and research

The progressive introduction of value-based reimbursements and the overall evolution of healthcare processes and hospitals is forcing the healthcare service industry, such as pharmaceutical companies and medical-technology suppliers, to incorporate VBM *value* in their strategy [3, 14, 58]. As a matter of fact, if not properly managed by healthcare services, VBM just raises new barriers to procurement and reimbursement, obliging companies to spend large amount of moneys and efforts in demonstrating the actual *value* of their product/services to health technology assessment (HTA) agencies, payors and insurers [3, 58]. In response to such a changed environment, healthcare service companies are embracing a new model of innovation, generating highly collaborative R&D ecosystems that comprise providers (usually hospitals). This allows i) the development of more focused technologies for the specific needs of a hospital and its regional network, and ii) the access to real-world patient outcome data, which are increasingly necessary for FDA/EMA and HTA agencies approvals [3, 14, 58]. In many cases, such precious data are traded-off for risk-sharing deals, in which procurement costs are proportional with the *value* actually generated by the innovation (new technology or drug) at the hospital involved in the deal (e.g. [48]).

As discussed in the introduction, the fire under the pot of healthcare VBM revolution are population ageing and the rise in chronic diseases, which to date account for over a third of the total healthcare expenditures at global level [1]. In 2019 alone, the number of people aged over 65 increased by 3.5% worldwide [59]. The reason for such a high drainage of resources for chronic diseases is not just their prevalence per se, but the fact that affected patients generally have multiple concomitant long-term conditions [60]. According to a 2017 report [61] US citizens with five or more chronic conditions are the 12% of population, but account for the 41% of total healthcare expenditure. Also, the 90% of US total healthcare expenditure goes for people with chronic or mental conditions [61]. Thus, chronic diseases management is the field where VBM process and system efficiency improvements can make a real difference. According to McKinsey Consulting projections, VBM implementation in chronic diseases could achieve savings of 9 to 16% on the total healthcare providers’ budget [1]. At a systemic level, this would lead to an average “*30% decrease in the average annual increase in national health expenditures*” [1]. This numbers explain why in the last decade such a high number of VBM and HTA studies and projects concentrated on chronic diseases. Among such diseases, we find interest in examining VBM application to stroke.

### Value-based medicine and stroke

The analysis of what is happening worldwide in the application of VBM principles to stroke is very instructive, since it provides a comprehensive picture of how the aforementioned trends are playing together, and it suggests an innovative macroeconomic perspective on what healthcare *value* can achieve in modern societies.

Stroke is a pathology characterized by: i) a complex in-hospital acute phase - where outcomes crucially depend on the timing of intervention - followed by a very long chronic phase, which requires extensive rehabilitation; ii) a high prevalence, since it affects about 200 people per 100,000 [62] and it ranks second as global cause of death, behind ischemic heart disease [63]; iii) a high economic and social cost, both for healthcare systems (about €21,000 per patient in 2010 [64]) and for informal caregivers. As such, much pressure has grown worldwide in optimizing the clinics, management and social costs linked with stroke.

By first considering the development of value-based reimbursement plans implementation, stroke is following the same pathway as other similar chronic or severely impairing pathologies. Various networks have been established worldwide to link the healthcare services provided by general practitioners (GPs) and consultants, hospitals and rehabilitation structures. A bright example is the Netherlands Heart Network (NHN), which operates in the Eindhoven area and works according to a VBM logic [65, 66]. Realizing how diverse was the management of patients with atrial fibrillation (a leading cause of stroke) by local GPs and cardiologists, NHN built an IT and managerial structure organized around the Catharina Hospital Eindhoven (the network hub) to harmonize and coordinate the clinical management of patients. The network links all relevant providers, here comprising GPs, consultants, nurses, ambulance service, thrombosis service, home care organizations, pharmacists, and diagnostic centres, with the aim of structuring a “total care delivery *value* chain” [66]. Data collection and analysis is shared with insurers, which represent the major payors in the Dutch system; at each step *value* is evaluated through PROMS defined by the Netherlands Heart Registration and the ICHOM system [67]. NHN, supported by a manufacturer’s sponsorship [68], provided innovative tools to consultants and at-risk citizens to promote early diagnosis and preventive care, established a network of outpatient clinics, and generated *value* by reducing costs and improving patient outcomes [65]. The economical sustainability of the network was achieved thanks to a funding model based on bundled payments agreed with insurers [66]. This experience was awarded with the ValueBased HealthCare Prize 2019 [66].

In parallel with value-based reimbursement, some value-based pricing experiences are recently gaining momentum. An example here is the recent deal between the US health insurer Harvard Pilgrim Health Care and the Pharma company Amgen for Repatha® (*Evolocumab*), a monoclonal antibody designed to decrease the risk of stroke and heart attacks by reducing circulating LDL cholesterol. In this case, the Pharma company took a risk-sharing approach, granting full rebate to the health plan if a patient suffers stroke or heart attack despite the regular assumption of Repatha®. This approach, already widespread in oncology [58], is now finding its way in other medical specialities, too. In this case, the actual risk-sharing approach is linked with a bet on the amount of *value* (i.e. savings) produced by the use of a very expensive category of drug and on the selection of the at-risk population that maximizes such *value*. It will be interesting in the future to measure the incremental *value* generated by such a deal, as compared with the standard situation of widespread prevention achieved though a less effective but extremely cheap class of drugs (statins) [69].

The need for ultrafast interventions at the stroke onset, together with very long-term rehabilitation after discharge, is forcing a boost in the IT capabilities of hospitals, in order to organize and exchange data in real-time with their local networks. In particular, the need for a fast differential diagnosis is driving the introduction of AI in the diagnostic process [16], both in the ambulance and at the ER, with four pieces of software for stroke diagnosis approved by FDA in 2018 only [16, 70]. Digital transformation and the large data sets now available in smart hospitals are also helping with the reduction of door-to-needle times inside hospitals, by allowing data-driven optimization of patient management. Rai and colleagues [49], for instance, demonstrated how the application of industrial optimization models, such as the Six-Sigma Motorola System, can reduce variability and improve outcome of stroke patients. In two recent experiences moreover, accurate data analysis helped reshape the pharmaco-economics [71] and the operations [72] of stroke patient management, respectively, with *measurable* impacts on *value*.

AI and Big data analysis also found application in assisting and improving standard neuroimaging (MRI and CT). Nowadays, numerous support-vector machine algorithms or Bayesian classifiers can detect, classify and segment stroke lesions on MRI/fMRI [73–76] or CT [29–31, 43, 77] brain images comparably with a trained expert. Similarly, IT improvements and AI have been employed to verify for each patient the clinical/economical suitability [78], the risk of side effects and the dose optimization of intravenous thrombolytic drugs [79–81]. Linked with the high costs of treatment and hospitalization, specialized literature is blossoming in a series of papers proposing AI- or artificial neural network-based algorithms to predict outcome or mortality at discharge or in the long run (most of them with a > 97% accuracy), based on the analysis of physiological parameters in the first 48 h from the event [82–84], or of standard medical images [43, 85, 86].

The encouraging decrease in stroke-related acute mortality seen worldwide comes together with a similar increase in the number of patients surviving with serious disabilities and requiring very-long-term rehabilitations before and after discharge [63]. This comes together with a situation of chronic lack of rehabilitation places in many OECD countries, such as Italy, where, according to recent estimations, only 20% of the necessary high-specialization rehab beds are available [87]. Logistic aspects (increased number of patients needing rehabilitation and chronic lack of beds), economical considerations (reduction in reimbursements, leading to shorter hospitalizations [88]) and recent data, supporting the early discharge of sub-acute stroke patients [89], are moving the prize for *value* toward smart solutions for home monitoring and tele-rehabilitation [12, 90]. Notwithstanding such techniques are still in their infancy, encouraging feasibility and cost-benefit studies are showing how, despite still inferior to standard in-person procedures [91], tele-rehabilitation is largely cheaper than standard approaches, and it expands the service to individuals who would not otherwise have received any [92].

**Fig. 1:**
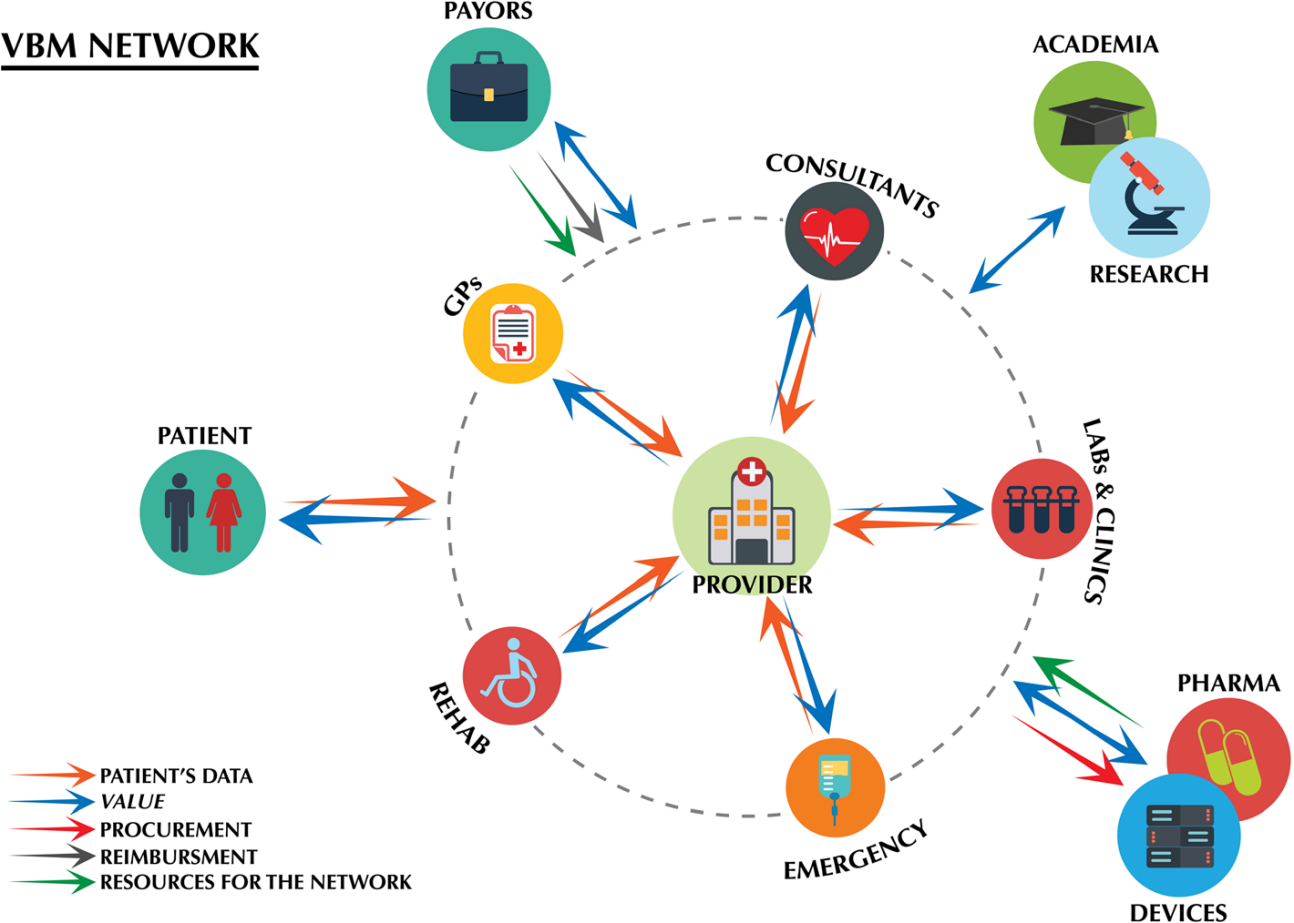
Graphic representation of the VBM network. Patients’ data concentrates toward the centre, the provider, which in turn redistribute value to all stakeholders. Resources for the network can derive from suppliers (pharma or medical devices companies) or from public or private payors. Procurement or reimbursement deals, together with exchange of value in the form of real-world data, keep suppliers and payors linked with the network. Icons designed by Makyzz - Freepik.com

By considering the aforementioned examples and applications altogether, one important lesson can be drawn on the application of VBM to a complex disease. The environments where providers, payors and suppliers produced *value* in a synergic way, as in the case of NHN, or the situations where technological innovations introduced real *value* to the system, as for AI diagnostics in the ambulance or tele-rehabilitation, are all resting on defined and well-structured networks (Fig. 1).

In the case of stroke, such digital and physical networks comprise hospitals and diagnostic centres (providers), general practitioners, consultants, emergency services and rehabilitation clinics, and possibly payors and service industries. The backbone of such networks is data. Hospitals need data from general practitioners, consultants and emergency services to improve the quality of their activities during hospitalization, particularly in VBM-reimbursement regimes. Also, to estimate *value* and refine processes, providers need post-discharge data, which in the vast majority of times derive from rehabilitation clinics and again, general practitioners and consultants. The more data accumulate toward the network hub, the hospital, the higher is the production of *value* for patients and society. This can take the form of i) medical performance for patients; ii) aggregated real-world data, good practices, savings on future reimbursements, et cetera, for healthcare service industries and payors; iii) algorithms, procedures and new knowledge for and from academia and scientific institutes. The existence of a network, and the availability of valuable real-world data, in fact, attracts healthcare service industries and private payors, which nurture the network with economic, technological or material resources. The same holds for Academia, which processes data to generate further social and economical *value*. At the same time, as already suggested by [3], what proves to be crucial for the maintenance of VBM networks is carefully designed procurement, since it binds the healthcare service industries, and their resources, to the network hub. Once such networks are established, and measurable clinical improvements are produced, virtuous circles get in place, promoting the redistribution of *value* for all stakeholders, in exchange for more data and further support to the network.

Watching these VBM networks through a macroeconomic lens, it seems to us that *value* is the currency exchanged among stakeholders in return from data. Clinical data accumulation toward the hub of the network generates *value* for patients, which *ab origine* are the owner of data. In a functioning VBM network, this redistribution of healthcare, social and economical *value* happens at multiple levels, encompassing all stakeholders and reaching a much wider audience of patients, as compared to the restricted number of them who actually provided the data. Hence, a functioning VBM network realizes a “circular economy” that pays patients with *value* for their clinical data, and, while growing, distributes social dividends to all stakeholders. As compared with the nowadays situation, where the flow of patients’ data and the one of clinical performance are indirectly correlated, mediated by necessity through academia and clinical research, the promise of value-based medicine is the construction of efficient economies of scale for *value*. Here, *value* is generated in a much more efficient way, and at all sites along the network. Similarly, *value* payback is disintermediated, since many actors in parallel generate and distribute *value*, and new knowledge can be built directly on each patient’s data. In our view, if healthcare providers resist the temptation to commoditize data, i.e. do not sell raw data to industries or payors, but maintain a central role in the exploitation of data for *value* production, VBM will achieve different goal in parallel. i) VBM will maximize *value* for patients, i.e. the clinical results patients value the most; ii) *value* maximization will save money to healthcare systems, both by hooking costs with effective healthcare benefits and by lowering operational costs; and iii) VBM will force providers to think in strategic terms, consolidating relationships with all stakeholders in networks.

## Conclusions

Our analysis of value-based medicine (VBM) led us to two key considerations: i) VBM is a key factor to make healthcare systems sustainable in the long-term, since it tackles the crucial productivity issue of the healthcare industry. ii) By establishing networks among stakeholders and by materializing the medical and social benefits for patients, payors and society, VBM can be the sought-after redistributive mechanism that compensates patients for the exploitation of their personal medical data.

The very quantitative-oriented approach to healthcare proposed by VBM is subject to some criticisms. According to some scholars, VBM’s approach disregards the value of the caring act and replaces trust in professionals with accountability, undermining solidarity as an essential aspect of medical acts [93]. Despite the argument deserves deep consideration, *scientification* of medicine, already started with evidence-based medicine, is in our opinion an essential ingredient of sustainability.

The ongoing introduction of VBM in healthcare systems rests upon two pillars: the generation of data-permeable networks, which keep *value* in circulation, and a value-based management of procurement, which ties healthcare service providers, and their resources, to the networks.

In this very moment, the ball is in the court of world-leading healthcare providers, which have the unique chance to define what *value* exactly is for each pathology. In the upcoming gold rush generated by this new economy, being the first in defining what the accepted data-of-interest and PROMS are for a disease will be a massive competitive advantage.

## Data Availability

Not applicable.
